# The effects of earthworm fibrinolytic enzyme on cervical cancer Caski cells based on CiteSpace and VOSviewer visual analysis

**DOI:** 10.1097/MD.0000000000046783

**Published:** 2026-01-02

**Authors:** Kaize Yuan, Yuehua Bai, Siyang Chen, Yiyao Shi, Miao Yu, Ke Zhang, Lihua Zhu, Shuying Li

**Affiliations:** a North China University of Science and Technology (Hebei Key Laboratory for Chronic Diseases), Tangshan, China.

**Keywords:** apoptosis, cervical cancer, earthworm fibrinolytic enzyme, migration, proliferation

## Abstract

**Objective:**

To investigate the effects of earthworm fibrinolytic enzyme (EFE) on proliferation, migration, and apoptosis of Caski cells.

**Methods:**

A visual analysis was conducted based on CiteSpace and VOSviewer using 257 studies related to EFE. The optimum concentration of EFE on Caski cells was screened by CCK-8 assay in vitro. The effects of EFE on Caski cell migration, tumorigenicity, protein expression, and apoptosis were determined by cell scratch assay, soft AGAR cloning assay, immunohistochemistry, and flow cytometry.

**Results:**

The antitumor activity of EFE has been a hot topic in recent years. EFE significantly inhibited the proliferation of Caski cells compared with the control group, and the difference was statistically significant (*P* < .0001). The scratch distance of low, medium, and high concentration cells was significantly increased compared with the control group in the cell scratch test, and showed a certain concentration dependence, and the difference was statistically significant (*P* < .05). The results of soft AGAR cloning showed that tumorigenicity of low, medium, and high concentration was significantly decreased compared with control group, and the difference was statistically significant (*P* < .001). Immunohistochemical assay showed that the positive rate of low, medium, and high concentration decreased significantly compared with the control group, and the expression of protein was downregulated. Flow cytometry showed that low, medium, and high concentration promoted apoptosis compared with the control group, and the difference was statistically significant (*P* < .01).

**Conclusion:**

EFE can inhibit the proliferation and migration of Caski cells and promote cell apoptosis.

## 1. Introduction

Cervical cancer, as the 4th most common malignant tumor among women worldwide, has approximately 604,000 new cases and 342,000 deaths each year,^[1]^ and it is particularly prevalent in developing countries.^[2]^ At present, clinical treatment is confronted with severe challenges such as large surgical trauma, strong resistance to radiotherapy and chemotherapy, low response rate of immunotherapy, and poor accessibility of targeted drugs.^[3]^ There is an urgent need to develop new antitumor drugs that are low-toxic, low-cost, and targeted. Against this backdrop, traditional Chinese medicine has demonstrated unique therapeutic advantages: its therapeutic concepts of “holistic regulation” and “strengthening the body’s resistance and eliminating pathogenic factors,” through multi-target and multi-pathway mechanisms of action, can not only effectively reduce the toxic and side effects of radiotherapy and chemotherapy and improve treatment tolerance, but also perform outstandingly in inhibiting tumor metastasis, reducing recurrence rates and improving the quality of life.^[4]^

Among the numerous traditional Chinese medicine resources with antitumor potential, earthworm (Pheretima), as a representative of traditional blood-activating and stasis-removing drugs, has attracted much attention due to its abundant resources and mature extraction process. Modern pharmacological studies have confirmed that the earthworm fibrinolytic enzyme (EFE) extracted from earthworms is a type of proteome with serine protease activity,^[5]^ and it has multiple functions such as anti-inflammation, anti-oxidation, anti-apoptosis, antibacterial, thrombolytic, and antitumor.^[6–9]^ Its antitumor potential is manifested in many aspects: In basic research, EFE can exert antitumor effects through multiple mechanisms such as inducing tumor cell apoptosis, blocking the cell cycle, and inhibiting angiogenesis^[10]^; in terms of combined therapy, EFE shows a synergistic effect with conventional chemotherapy drugs and can alleviate the toxic and side effects caused by chemotherapy drugs^[11]^; it is particularly notable that EFE still maintains a good inhibitory activity on chemotherapy-resistant tumor cells,^[12]^ which provides a new possibility for solving the clinical drug resistance problem. This research not only provides important clues for the development of new anti-cervical cancer drugs, but also opens up a new direction for the modernization research of traditional Chinese medicine.

**Figure 1.**
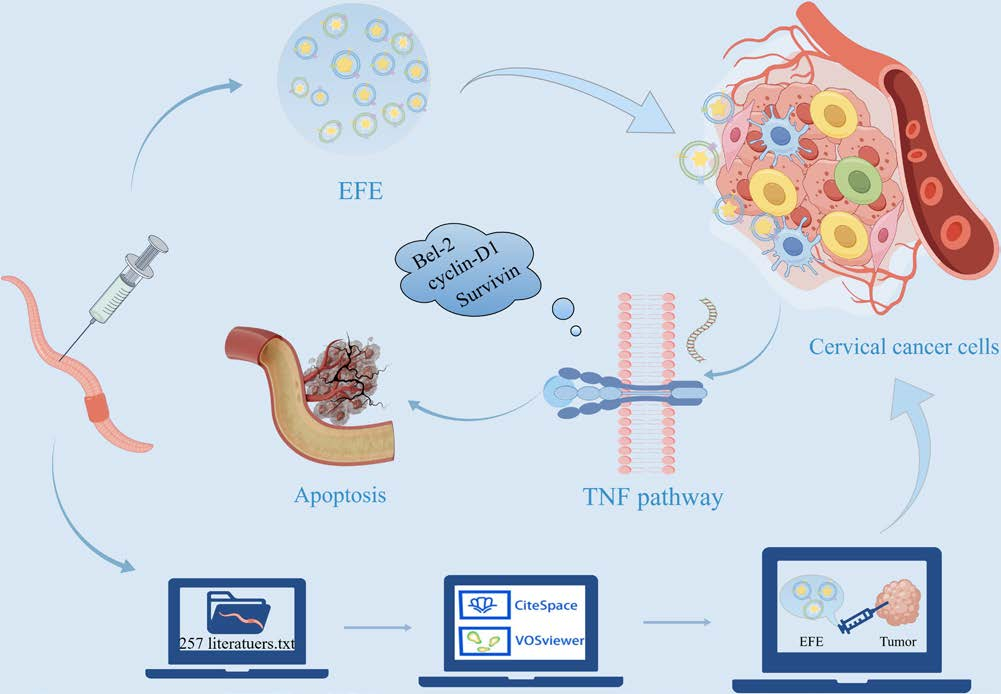
Abstract diagram by Figdraw software.

In order to further explore the antitumor mechanism of EFE, this study based on CiteSpace and VOSviewer conducted a visual analysis of the research popularity and development trend of 257 studies related to EFE. The effect of EFE on the proliferation and apoptosis of Caski cells of cervical cancer was investigated through in vitro experiments, and the mechanism of EFE’s anti-cervical cancer was analyzed to provide theoretical basis for clinical treatment of cervical cancer (Fig. 1).

## 2. Materials and methods

The Caski cells were preserved and provided by Hebei Key Laboratory of Chronic Diseases, North China University of Science and Technology.

*The main reagents and instruments*: CCK-8 kit, immunohistochemical kit, DAB chromogenic agent, DMEM culture solution, serum, rabbit anti-BCL2, cyclin D1, survivin monoclonal antibody, and HRP labeled sheep anti-rabbit IgG, CO_2_ cell incubator were purchased from Beijing Solaibao Technology Corporation, Beijing, China. Flow cytometer BIO-RAD products; fluorescent microscope products of Nikon Corporation (Japan). EFE dry powder was donated by Professor Zhao Xiaoyu of Hebei University.

## 3. Methods

### 3.1. The visual analysis of earthworm fibrinolytic enzyme related research using CiteSpace and VOSviewer

#### 3.1.1. The criteria for inclusion and exclusion of articles

Total 619 articles from January 1, 1991 to July 21, 2024 were screened in Web of Science (WOS), PubMed, and Scopus database. Among them, 215 were published in WOS, 143 in PubMed, and 261 in Scopus.

The following formula is used as a search formula in WOS and PubMed:

(1) TS = vermis kinase OR EFE OR boluokenu OR earthworm fibrinolytic enzyme component A protein, Eisenia fetida.
(2) TS = EFE component A protein, Eisenia fetida.

In Pubmed:

(1) Vermis kinase OR EFE OR boluokenu OR earthworm fibrinolytic enzyme component A protein, Eisenia fetida.
(2) EFE component A protein, Eisenia fetida.

In Scopus:

TITLE-ABS-KEY (vermis AND kinase) OR TITLE-ABS-KEY (earthworm AND fibrinolytic AND enzyme) OR TITLE-ABS-KEY (boluoke) OR TITLE-ABS-KEY (earthworm AND fibrinolytic AND enzyme AND component AND a AND protein, AND eisenia AND fetida) OR TITLE-ABS-KEY (EFE AND component AND a AND protein, AND eisenia AND fetida).

The duplicated articles, the conference abstracts, book chapters, conference proceedings papers, and other irrelevant articles were deleted after sorting and statistics of the search results. Two hundred fifty-seven valid articles were obtained finally.

#### 3.1.2. The visual analysis using CiteSpace6.2.R6 and VOSviewer1.6.20

Bibliographic management software EndNote (Thomson Corporation, Stanford) was used to sort out this paper, and Citespace6.6.R6 and VOSviewer1.6.20 were used to analyze.

The number of published papers, countries, journals, and keywords of all articles were analyzed using CiteSpace6.2.R6 and VOSviewer1.6.20 through bibliographic management software EndNote, and so as to understand the international research popularity and future development trend of EFE.

### 3.2. The effects of EFE treatment on Caski cells were detected in vitro

#### 3.2.1. The effect of EFE treatment on Caski cell viability was detected by CCK-8 method

Caski cells were routinely cultured for approximately 48 hours and collected when the cells were in the logarithmic growth phase. Adjust the density of Caski cells to 1 × 10^3^ per milliliter and add them to each well of a 96-well plate, with approximately 100 µL added to each well. Subsequently, these cells were continued to be cultured in a cell incubator at 37 °C with 5% carbon dioxide. The experimental groups included a blank group (no cells), a control group (treated with drug-free culture medium), and multiple experimental groups with different mass concentrations of EFE (1, 2, 3, 4, 5, 6, 7, 8, 9, and 10 uku/mL). When the cell confluence reached 60%, different concentrations of EFE and control substances were added to each well. Each EFE concentration treatment group contains 6 repeat Wells. At 24 hours, 48 hours and 72 hours after EFE treatment, 10 µL of CCK-8 solution were added, respectively, and the culture continued for 1 hour under 37 °C and away from light. Finally, the absorbance value at 450 nm was detected by an microplate reader.

Cell vitality was calculated according to the formula: cell vitality = (OD of detection group with different EFE concentration treatment ‐ OD of blank control with medium group)/(OD of cell control group ‐ OD of blank control with medium group). At the same time, the cell morphology was observed under the microscope, and the cell area was calculated and mapped. The parallel experiment was performed 3 times. the experimental results were statistically analyzed by Graphpad software (San Diego). According to IC50 = 5.283, the subsequent experiments adopted 3 uku/mL, 5 uku/mL, and 7 uku/mL as low, medium, and high drug concentrations for the subsequent experimental groups.

#### 3.2.2. The Caski cell wound scratch assay

Caski cells at logarithmic growth stage were collected, the cell density was diluted to 1 × 10^5^ cells/mL using serum-free DMEM medium, and the cells were inoculated in 6 well plates with 2.0 mL cells per well.

Linear scratches were made on the bottom cells of the 6-well plate with a sterile pipetting tip after incubating the cells for 24 hours, and the cells were cleaned with PBS for 3 times, and 2 mL DMEM medium containing EFE concentration about 0, 3 uku/mL group, 5 uku/mL group, and 7 uku/mL without serum was added to each well, respectively, and continuous cultivation 48 hours. Caski cells in 6 well plate was observed under inverted microscope and taken photos at 0 hour and 48 hours, respectively. The scratch area was measured using ImageJ software (National Institutes of Health). The migration distance is calculated according to the formula, migration distance = scratch area/height. Parallel experiments were conducted for 3 times, and the experimental results were statistically analyzed by Graphpad software.

#### 3.2.3. The cell proliferation was detected by soft AGAR cloning

Substrate AGAR preparation: 20% DMEM medium containing 0.7% sterile AGAR was added to a 12-well plate at an amount of 1 mL/well until cooled and solidified.

##### 3.2.3.1. Upper AGAR preparation

Cell density was adjusted to about 1 × 10^7^ cells/mL using DMEM medium containing 20% serum, and the same amount of 0.7% sterile AGAR was added, mixed well. Which was divided into 4 groups including 0, 3, 5, and7 uku/mL EFE, respectively, and added to 12-well plates (1 mL/well), each group of EFE was provided with 3 compound wells, 0.5 mL complete culture solution containing the above corresponding concentration EFE was added each well after the upper AGAR cooling and solidification. Above 12-well plate was cultured at 37 °C in 5% CO_2_ incubator, the culture medium on the AGAR surface was changed every 2 to 3 days. These cells were cultured continuously for 14 days. The clones in the soft AGAR were observed every day, and the number of clones was counted. The cell division was taken as one clone for 3 times, and the clone formation rate was calculated according to the following formula: clone formation rate (%) = (numbers of clones/number of counted cells) × 100%. At the same time, the clone area was observed and photographed under the microscope. The parallel experiments were conducted in 3 times, and the experimental results were statistically analyzed using Graphpad software.

#### 3.2.4. Immunohistochemical staining

(1) Preparation of Caski cell slides: 10 small aseptic cover slides were placed into each well of the 12 well plate, 2 mL prepared cell suspension (cell concentration 5 × 10^3^ living cells/mL) was added in each well, and the cell culture medium was replaced when the cell confluence reached 60%. The cell culture medium was replaced with cell culture medium containing 3, 5, 7 uku/mL EFE, and using cells without EFE as the control, and each concentration group had 3 compound wells. The cells were cultured continuously for 48 hours.
(2) Fixed cells: remove the cover slide covered with cells, wash it with PBS twice, add paraformaldehyde and fix it at room temperature for 15 minutes, rinse with PBS buffer 3 times for 3 minutes each time.
(3) Block endogenous peroxidase: 3% hydrogen peroxide solution was added to the cover glass of crawling cells and incubate at room temperature for 25 minutes away from light. The cells were washed using PBS buffer in a shaker 3 times for 5 minutes each time to eliminate endogenous peroxidase activity.
(4) Incubation of primary and secondary antibodies: primary antibodies bcl2, cyclin D1 and survivin were added to cells in the slide in each group and incubated in a wet box at 4 °C overnight. These cells were washed 3 times with PBS buffer for 5 minutes each time. Appropriate amount of goat anti-mouse IgG (secondary antibody) polymer was added to cells and incubated at 37 °C for 20 minutes. Then cells were washed 3 times with PBS buffer for 5 minutes each time.
(5) DAB color development: the diluted DAB was added to the cells and colored away from light for 5 minutes, and quickly washed cells with tap water to terminate coloration. These cells were re-dyed for 3 minutes using hematoxylin, and washed with tap water, and differentiated a few seconds using 1% hydrochloric acid ethanol solution, and washed with tap water, and returned blue using 10% ammonia water, and quickly rinsed with running water.
(6) Observation: the cells in 6-well plate were observed under the microscope and photographed by scanning imaging system for statistical analysis.

#### 3.2.5. Flow cytometry

Caski cells were cultured conventionally and collected during logarithmic growth phase. The cell concentration was regulated to 5 × 10^5^ cells/mL, and inoculated into 6-well plate with 2 mL for each well. 3, 5, 7 uku/mL EFE were added into each well of 6-well plate, after continuous cultivation for 12 hours. These cells were washed twice using PBS, and digested with EDTA-free pancreatic enzymes after continuous cultivation for 24 hours, and centrifuged at 2000 rpm for 5 minutes and collect cells. Then these cells were washed twice with PBS again, and centrifuged at 2000 rpm for 5 minutes, and the cell concentration was adjusted to 5 × 10^5^ cells/mL. 500 μL binding buffer and 5 μL annexin Ⅴ-EGFP were added to these cells and mixed them, and mixed with 5 μL propidium iodide (PI). These cells were detected by flow cytometry after away from light for 15 minutes at room temperature.

#### 3.2.6. Statistical analysis

All data are expressed as mean ± standard deviation (x ± s). The graph is constructed by GraphPad Prism 9.5.1 software, statistical analysis is conducted by IBM SPSS Statistics 27 software, and the significance of the differences among the groups is determined by one-way variance test, and the *T* test analysis is conducted by Student *t* test (ns, not significant; *P < .05, **P < .01, ***P < .001, ****P < .0001).

## 4. Results

### 4.1. Visual analysis of EFE related articles using CiteSpace and VOSviewer

#### 4.1.1. Publication quantity analysis

**Figure 2.**
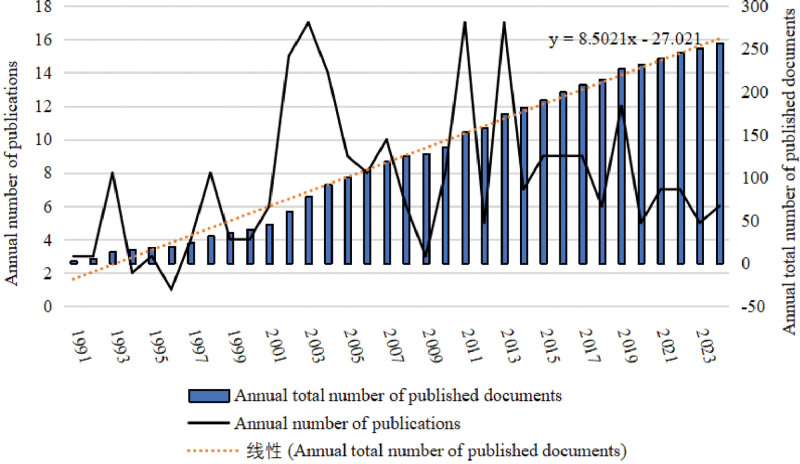
The number of articles published in each year.

The number of published articles related to EFE has fluctuated in the past 30 years according to the number of published papers in each year (Fig. 2), but the range of change is not large, therefore, the total number of publications increased linearly.

#### 4.1.2. Analysis of national cooperation network

**Figure 3.**
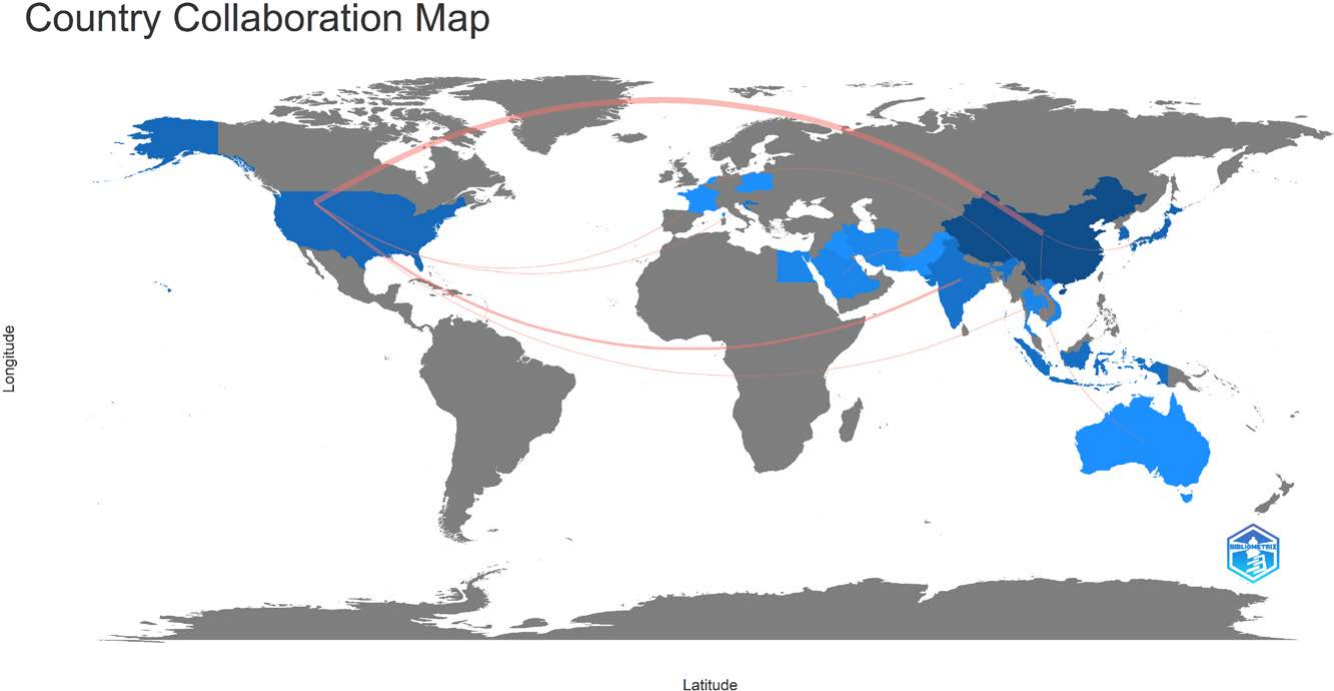
National cooperation network.

The darker the blue color, the more research the country has done in this area in the country Collaboration Network (Fig. 3). As can be seen from Figure 3, the cooperation between countries is mainly concentrated in the northern Hemisphere, especially in Asian countries, and China’s blue color is the deepest, indicating that China has more research in this field, which is inseparable from China’s long traditional Chinese medicine culture. In addition to the more concentrated blue areas, there are countries with more gray areas in the figure, which indicates that the current research in this field is more concentrated, the scope of influence is small, and the international research enthusiasm in this field is not high.

#### 4.1.3. Periodical dual mapping analysis

**Figure 4.**
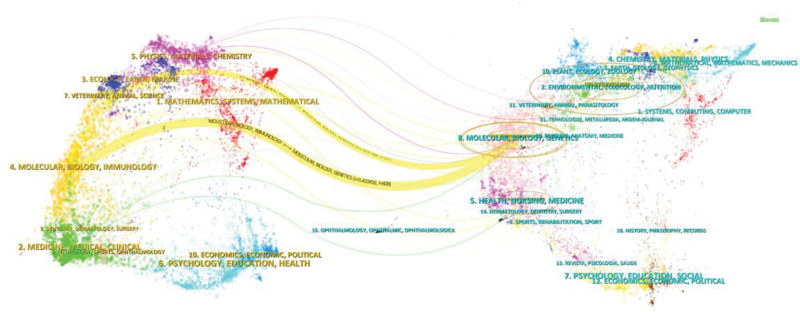
Periodical overlay double map.

The left side represents the different sets of the types of journals cited, the right side represents the sets of the types of journals cited, and the curve is the citation line for the complete presentation of the context of the citations in the double mapping superposition diagram of journals (Fig. 4). The prominent yellow color indicates that articles published in the fields of medicine, biology, immunology, and pharmacy are cited in journals related to biology, immunology, ecology, and mathematics, which reflects the influence of EFE in many fields such as medicine and ecology, attracting more researchers to explore in this field, and laying a foundation for the exploration of anti-inflammatory, thrombolytic, anticancer, and other mechanisms of action in clinically relevant diseases.

#### 4.1.4. Keyword analysis

##### 4.1.4.1. Keyword overlay analysis

**Figure 5.**
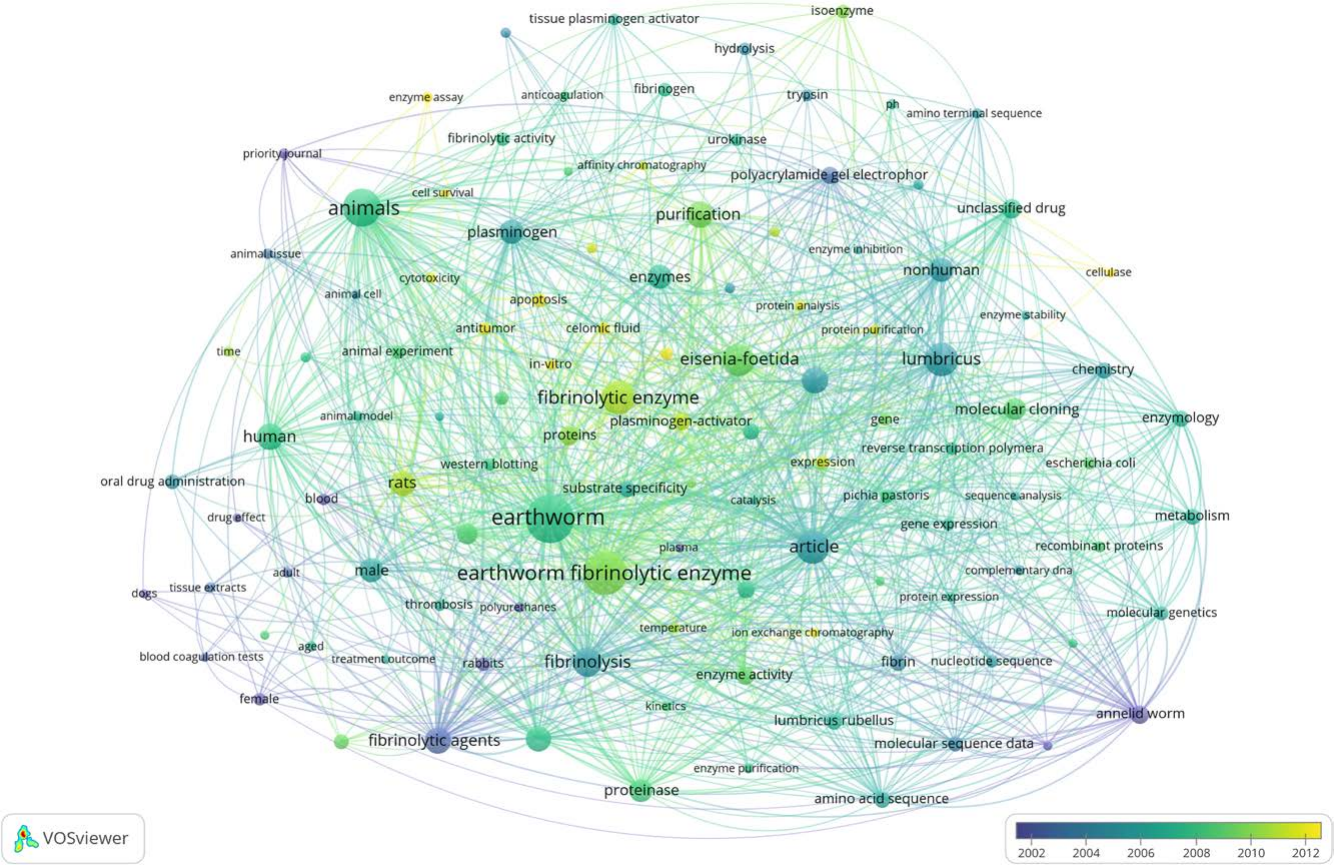
Keyword overlay diagram.

Keywords represent the central topic of an article, and the keyword overlay graph can quickly capture the research hotspots in a certain field in a certain period of time. The keywords were summarize using VOSviewer (Fig. 5). The color of keywords in the figure changes with time, and the size of nodes increases with the increase of frequency.

The most frequent keywords include earthworm, earthworm fibrinolytic enzyme, eisenia-foetida, etc. These keywords were mostly concentrated in the articles from 2004 to 2010, and the researches at this stage were mostly related to the keywords such as anticogulation, western blotting, purification, etc. The composition of plasminase varies among different species of earthworms. The fibrinolytic activity and the ability of inhibiting platelet aggregation against thrombosis were explored by purification and determination of EFE isoenzymes.^[13]^ Its broad substrate specificity and inducible substrate high reactivity are used for antithrombotic therapy and other potential diseases.^[7]^ In recent years, the key words are mostly related to antitumor, apoptosis and cytotoxicity, although the frequency is not high, it also indicates that the research focus in this field has been expanded from the antithrombotic properties of EFE to antitumor, anti-oxidation properties.

##### 4.1.4.2. Keyword cluster analysis

**Figure 6.**
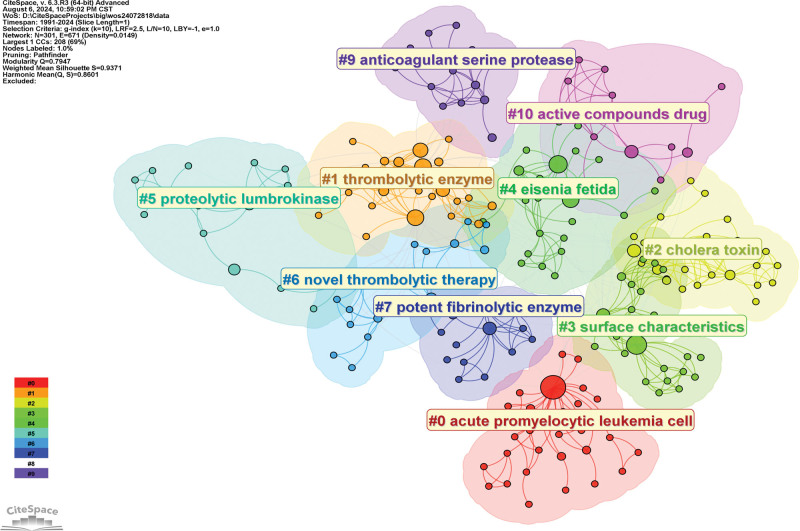
Keyword clustering diagram.

The keyword clusters studied with EFE are divided into 10 main clusters (Fig. 6) using the statistical analysis technique of relatively homogenous groups, and different clusters represent the general research directions of different fields or disciplines. Among them, #1, #4, #6, #7, and #9 are targeted at fibrinolytic activity and antithrombotic properties; #2, #3, and #5 target their intestinal absorption and anti-inflammatory properties; #0 and #10 target antitumor activity.

Keyword clustering pays more attention to the fields involved in the research content and its impact on a certain field compared with the above keyword superposition analysis. This cluster diagram summarizes the effect of EFE as an important active substance that activates fibrinolysis to achieve antithrombotic and anti-fibrosis^[6]^ It can be used to prevent ischemic diseases such as cardiovascular and cerebrovascular diseases and reperfusion injury by reducing blood viscosity,^[14]^ and EFE has anticoagulant, antioxidant, antibacterial, and antitumor activities.^[15,16]^

### 4.2. Effects of EFE on proliferation, migration, protein expression, and apoptosis of Caski cells

#### 4.2.1. Experimental results of CCK-8

**Figure 7.**
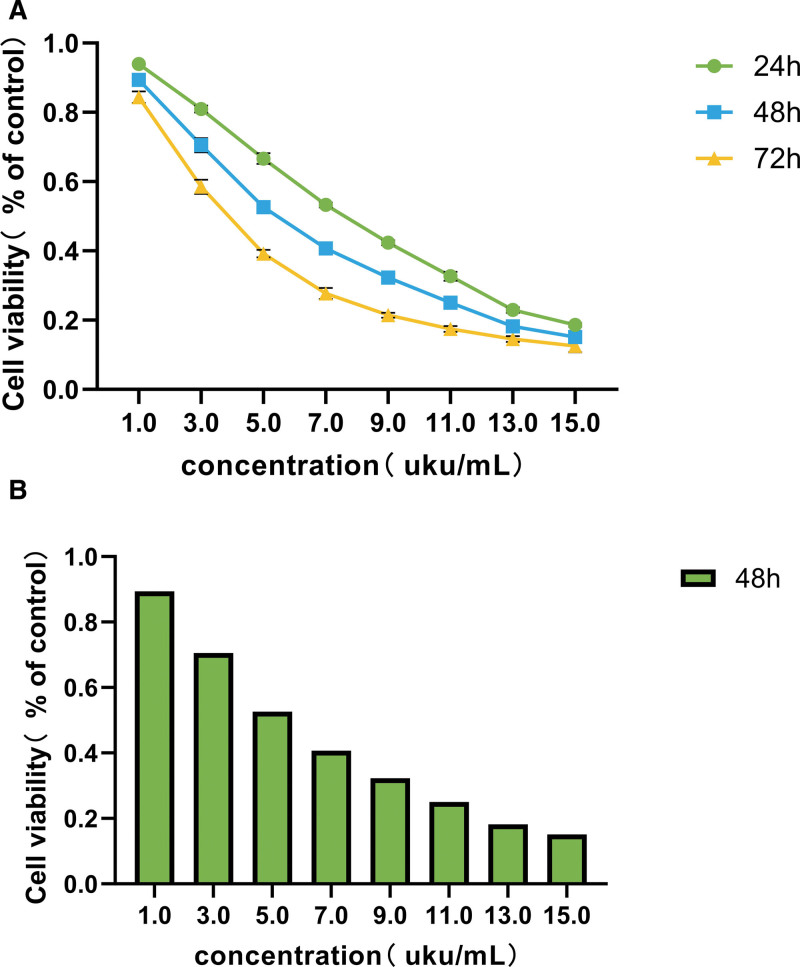
The effect of EFE on Caski cell activity was detected by CCK-8 assay. (A) The effect of EFE at different time and concentration on Caski cell activity. (B) The effect of different concentration EFE treatment Caski cell activity for 48 hours. EFE = earthworm fibrinolytic enzyme.

The results of CCK-8 showed that EFE with different concentrations could reduce the activity of Caski cells at 24 hours, 48 hours, and 72 hours, respectively (Fig. 7A), and the cell activity decreased with prolonged time, and the cell activity decreased significantly with the increase of EFE concentration (Fig. 7B). There was significant difference between each concentration group and control group (*P* < .01). IC50 = 5.283. The low, medium, and high concentrations of each subsequent experimental group were set to be 3, 5, and 7 uku/mL according to IC50, respectively.

#### 4.2.2. Morphological changes of Caski cells in each group

It was observed under the microscope that the number of cell death increased with the increase of EFE concentration in each group compared with the control group after EFE treatment on Caski cells for 48 hours.

**Figure 8.**
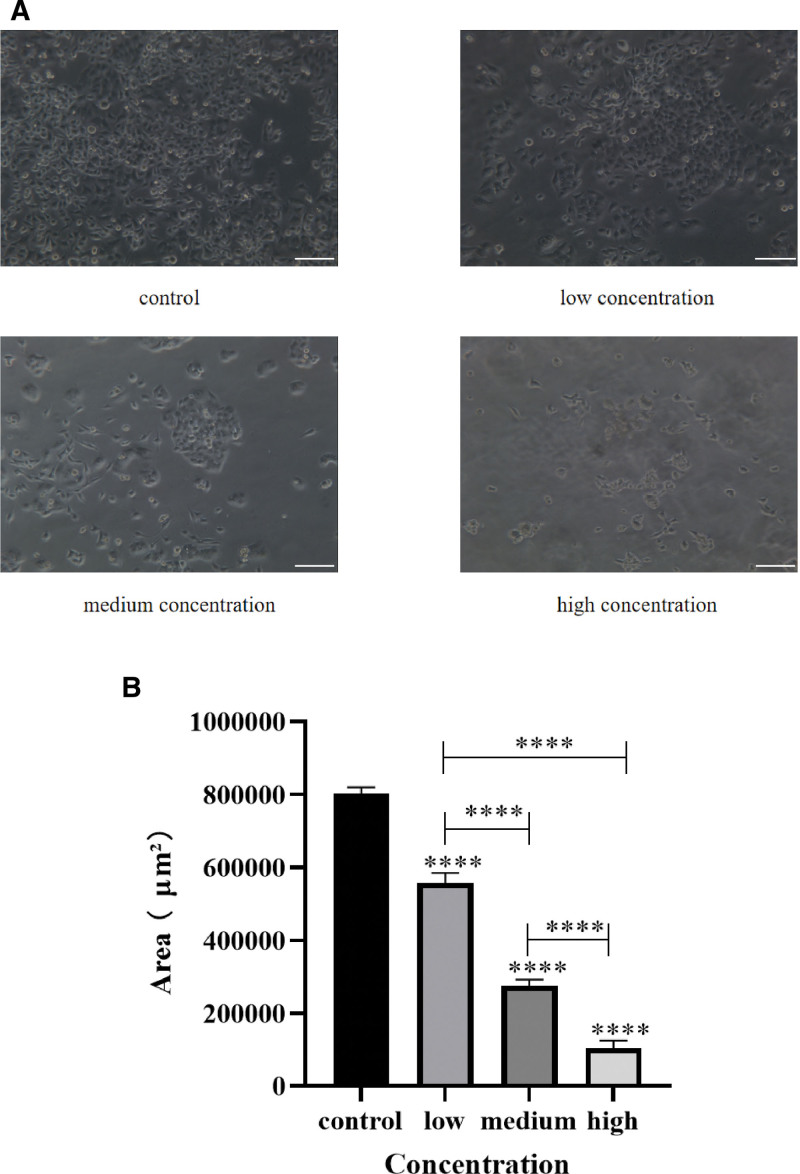
Morphological observation and statistical analysis of Caski cells in low, medium, and high concentration EFE treatment groups. (A) The morphology of Caski cells in each group treated with low, medium, and high concentration EFE for 48 hours (scale: 50 μm). (B) The living cell area statistics of Caski cells in each group treated with low, medium, and high concentration EFE for 48 hours. EFE = earthworm fibrinolytic enzyme.

The morphology of the cells gradually changed from the original long spindle shape to a round stack, wrinkled, the connections between cells decreased, and some cells fell off, However, there were still a few surviving cells in the high concentration group (Fig. 8A). Meanwhile, the statistical results of the area for surviving cells showed that the area of surviving cells decreased with the increase of EFE concentration (Fig. 8B), and the difference was statistically significant compared with the control group (*P* < .01).

#### 4.2.3. Cell scratch test results

**Figure 9.**
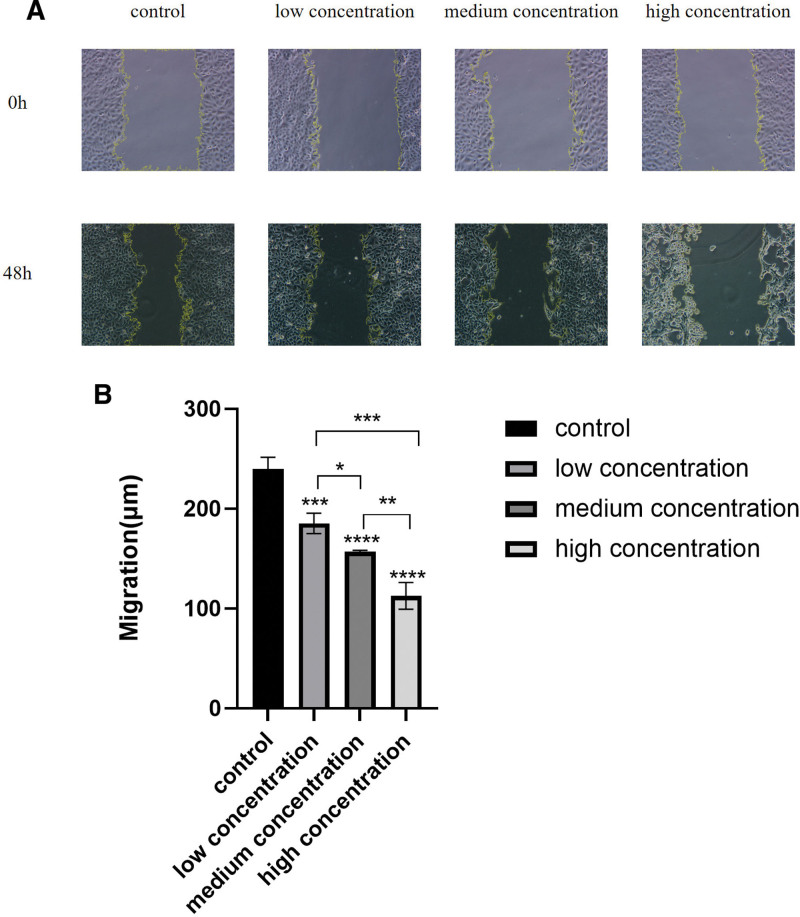
The result of 48 hours scratch test of Caski cells in each group treated by EFE. (A) The cell migration in different concentrations of EFE treated for 48 hours through scratch test (the scale: 50 μm). (B) Statistical analysis of cell migration in different concentrations of EFE treated in 48 hours through scratch test. EFE = earthworm fibrinolytic enzyme.

The cell scratch test results showed that EFE treated cells for 48 hours could significantly inhibit Caski cell migration, and cell growth showed a dose-dependent trend, and the higher the concentration, the greater the inhibitory effect on cells (Fig. 9A). Statistical analysis showed that the difference was statistically significant (*P* < .01) (Fig. 9B).

#### 4.2.4. The results of soft AGAR cloning experiment

**Figure 10.**
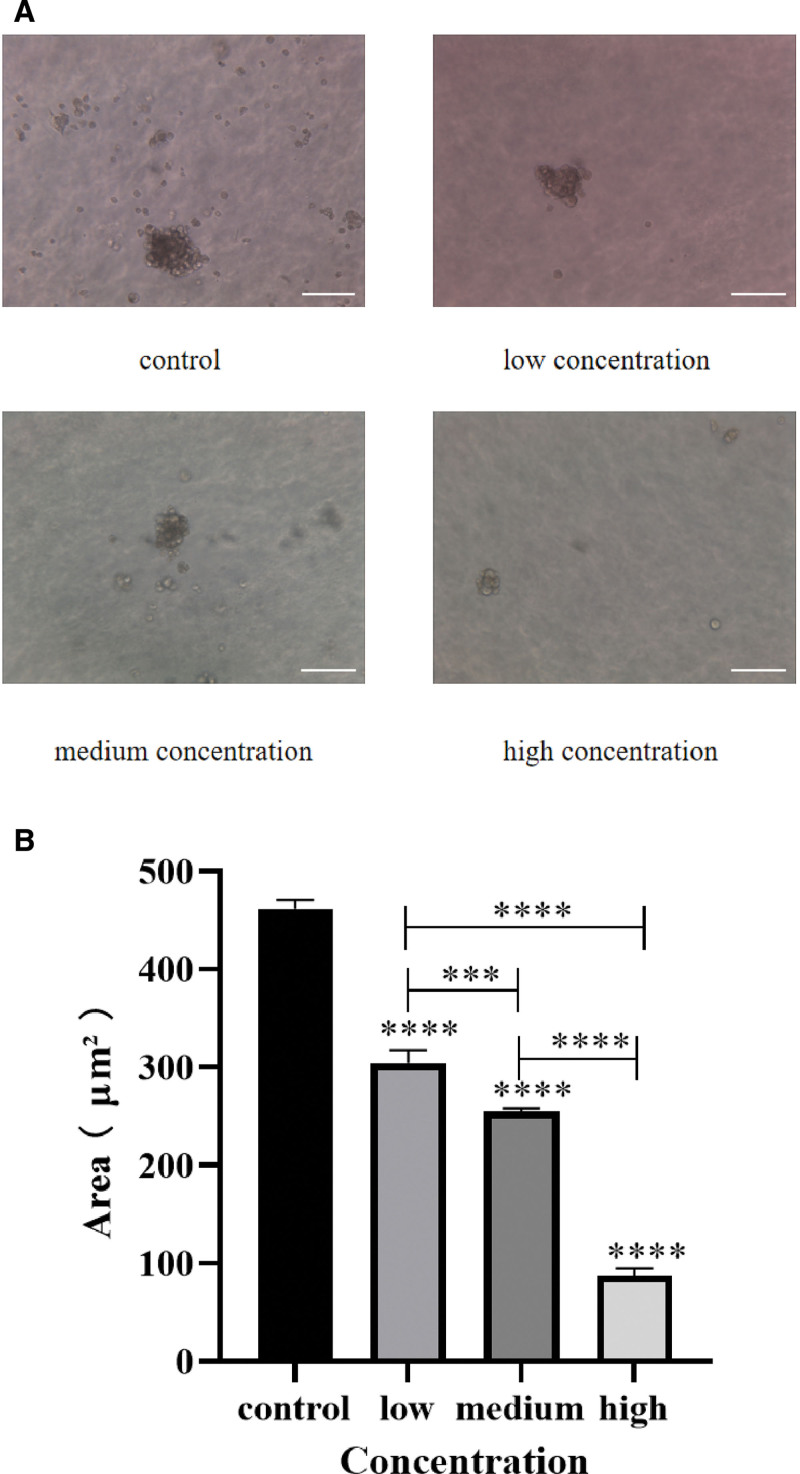
The effect of low, medium, and high concentration EFE on Caski cell proliferation was detected by soft AGAR cloning. (A) The effect of low, medium, and high concentration EFE on Caski cell proliferation was detected by soft AGAR cloning, the scale: 50 μm. (B) Statistical analysis of low, medium, and high concentration EFE on Caski cell proliferation ability. EFE = earthworm fibrinolytic enzyme.

Microscopic observation showed that the cells in the control group and the low-dose group of EFE had formed clones on the 7th day of cell culture, while cells in the medium-dose group and high-dose group of EFE had not formed clones. The cell clones for the control group and the low-dose group of EFE were significantly larger, and the cell clones for the medium-dose group and high-dose group were also observed at the 11th day. The clone size increased and the clone morphology was typical on the 14th day of culture (Fig. 10A). The clone formation rates were 89.0% (178/200), 43.5% (87/200), 24.5% (49/200), and 17.5% (35/200), respectively. Statistical analysis showed that: there was a statistically significant difference in cell clonal formation among all groups (χ2 = 240.5, *P* < .05), indicating that EFE significantly inhibited Caski cell proliferation, and the difference was statistically significant (*P* < .001), with a dose-dependent trend (Fig. 10B).

#### 4.2.5. Immunohistochemical staining results

**Figure 11.**
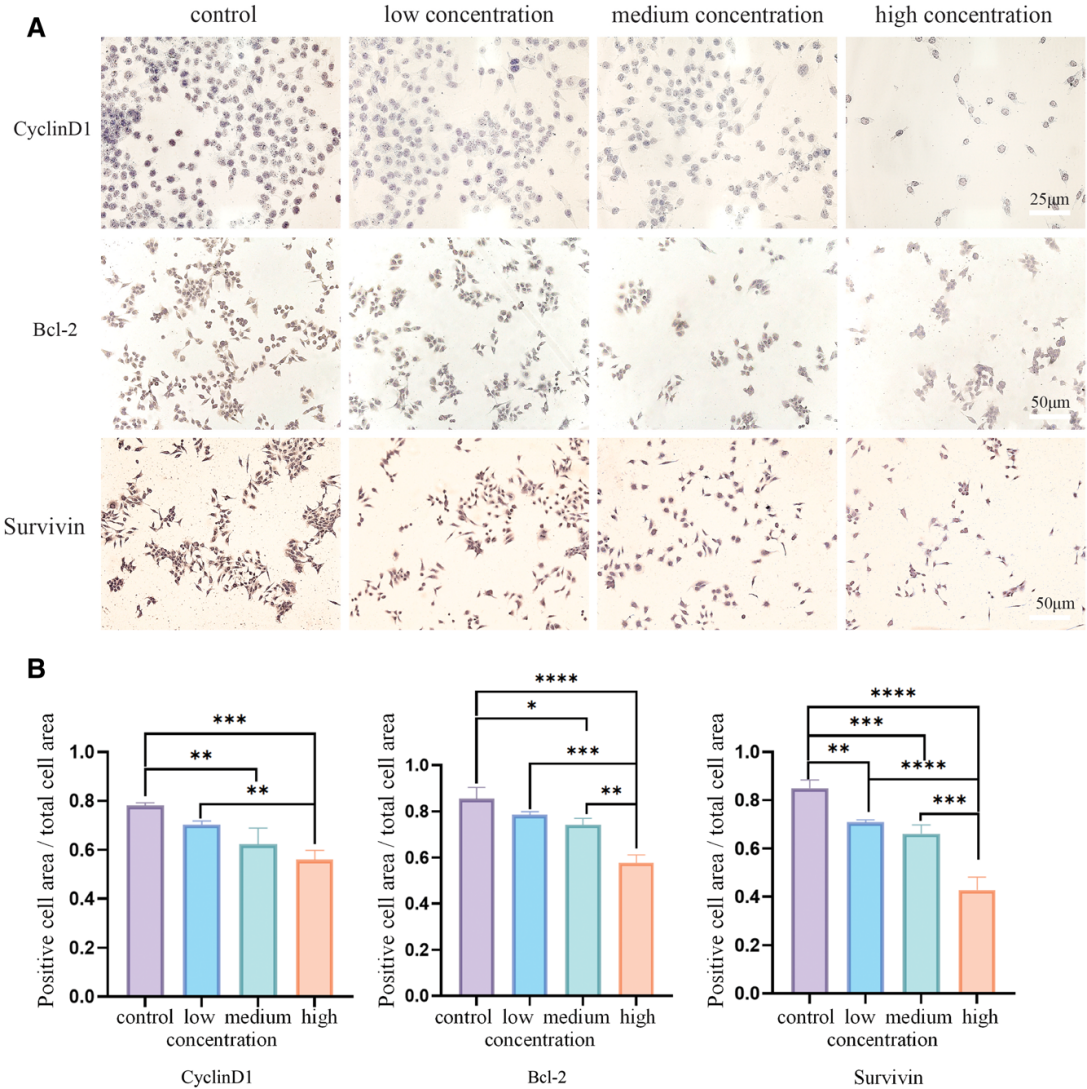
The effect of low, middle, and high concentration EFE groups on protein expression in Caski cells was detected by immunohistochemistry. (A) The effect of low, middle, and high concentration EFE groups on protein expression in Caski cells was detected by immunohistochemistry. (B) Statistical analysis of low, medium, and high concentration EFE on Caski cell protein expression. EFE = earthworm fibrinolytic enzyme.

Immunohistochemical staining results showed that the positive expression of Bcl-2 was all located in the cytoplasm, and the DAB staining showed brown-yellow color, while the negative result showed no color. After hematoxylin counterstain, the nucleus showed a counterstain blue color (Fig. 11A). EFE at various concentrations could reduce the expression of bcl2 compared with the control group Caski cells, and the expression degree decreased significantly with the increase of EFE concentration. The difference was statistically significant (*P* < .05) (Fig. 11B).

The positive expression of cyclin D1 was all located in the nucleus, and the DAB staining showed brown-yellow color, while the negative result showed no color. After hematoxylin counterstain, the nucleus showed a counterstain blue color (Fig. 11A). EFE could reduce cyclin D1 expression compared with control group cells, and the expression degree decreased significantly with the increase of EFE concentration. The difference was statistically significant (*P* < .05) (Fig. 11B).

Immunohistochemical staining of survivin is based on the presence of clear brown-yellow particles in the cytoplasm and/or nucleus as positive cells. Nuclei show a counterstain blue color after hematoxylin counterstain (Fig. 11A). EFE could reduce survivin expression compared with the control group cells, and the expression degree decreased significantly with increasing EFE concentration. The difference was statistically significant (*P* < .05) (Fig. 11B).

#### 4.2.6. Results of flow cytometry

Apoptosis was detected by annexin V-FITC/PI cell staining. In the early apoptotic cells, due to membrane overturning, the phosphatidylserine annexin VFITC staining in the membrane showed annexin V-FITC single positive. In the late apoptotic cells, due to membrane rupture, PI entered the cell and stained the nucleus. The cells showed double positive of PI and annexin V-FITC.

**Figure 12.**
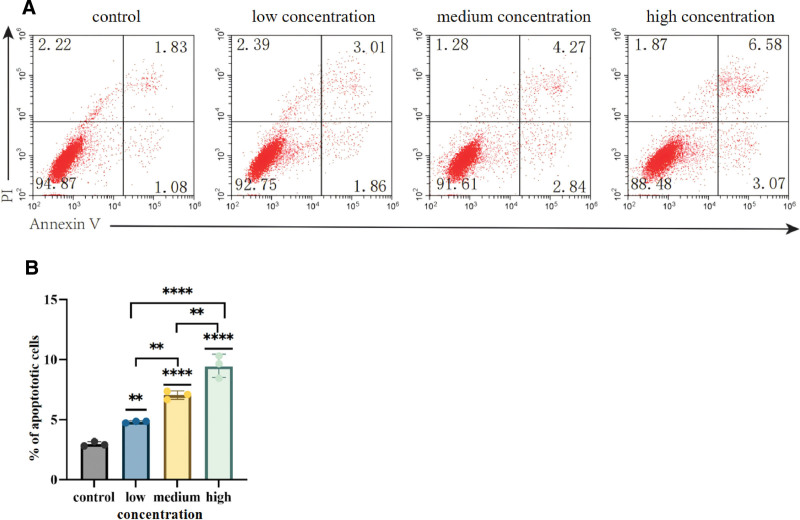
The effect of Caski cell apoptosis in low, medium, and high EFE groups were detected through flow cytometry. (A) The effect of Caski cell apoptosis in low, medium, and high EFE groups were detected through flow cytometry. (B) Statistical analysis of low, medium, and high concentration EFE on Caski cell apoptosis. EFE = earthworm fibrinolytic enzyme.

Flow cytometry results showed that the apoptosis rate of the control group was 2.91%, the apoptosis rate of the low concentrate group was 4.87%, the medium concentrate group was 7.11%, and the high concentrate group was 9.65% (Fig. 12A), the apoptosis rate of all groups was significantly increased with the increase of EFE concentration (Fig. 12B). These results indicated that EFE could enhance the apoptosis of Caski cells. The apoptosis rate showed an increasing trend compared with low concentrate group, medium concentrate group and high concentrate group. Statistical analysis showed that the apoptosis rate also increased with the increase of EFE concentration, and the difference was statistically significant (*P* < .05).

## 5. Discussion

EFE is a water-soluble protein extracted from the traditional Chinese medicinal material earthworm.^[17]^ As a bioactive substance with significant fibrinolytic activity, EFE has been developed into an oral preparation in East Asia and is widely used in the prevention and treatment of thrombotic diseases.^[13]^ Its mechanism of action is mainly to reduce blood viscosity and play an important role in the prevention and treatment of cardiovascular diseases and the protection against ischemia–reperfusion injury.^[18]^ In recent years, with the deepening of research, the potential value of EFE in the field of antitumor has attracted increasing attention, but its specific antitumor mechanism still remains to be clarified.

Through bibliometric analysis, we found that the anticancer activity of EFE has become a current research hotspot. It is worth noting that for the 4 major malignant tumors with a high incidence among Chinese women (breast cancer,^[19]^ colorectal cancer,^[20]^ lung cancer,^[21]^ and cervical cancer), EFE has achieved certain breakthroughs in mechanism research and clinical application progress in the first 3 cancers. However, there are still obvious gaps in the research on the mechanism of action of EFE on cervical cancer. This research gap provides an important opportunity for this subject. This study innovatively explores the effects of EFE on the proliferation and apoptosis of cervical cancer cell Caski, aiming to provide new experimental evidence and theoretical basis for clarifying the mechanism of EFE’s anti-cervical cancer effect.

The antitumor effect of EFE is closely related to the process of apoptosis it induces.^[8]^ Apoptosis, as a highly ordered form of programmed cell death, plays a key role in maintaining tissue homeostasis, and its abnormal regulation is an important characteristic of malignant tumors.^[22]^ Studies have shown that EFE may exert antitumor effects by regulating 2 major apoptotic signaling pathways: the exogenous death receptor pathway and the endogenous mitochondrial pathway. In the exogenous apoptotic pathway, after the death receptor (such as FAS/TNFR) binds to the corresponding ligand, caspase-8 is activated through the death-inducing signaling complex.^[23]^ Depending on the cell type, this pathway can be divided into 2 modes: in type 1 cells, caspase-8 directly activates downstream effector molecules; in type 2 cells, the death signal needs to be transduced to mitochondria through the Bid protein.^[24]^ The endogenous apoptotic pathway (also known as the mitochondrial pathway) is mainly regulated by the Bcl-2 protein family.^[25]^ This family contains antiapoptotic members (such as Bcl-2, Bcl-xL) and pro-apoptotic members (such as Bax, Bak),^[26]^ mediating the release of cytochrome c by regulating the permeability of the outer mitochondrial membrane.^[27]^ The released cytochrome c combines with APAF-1 to form apoptotic bodies, which in turn activates caspase-9.^[28]^ Antiapoptotic Bcl-2 protein inhibits this process by binding to the BH3 domain of pro-apoptotic proteins.^[29]^ EFE exerts a broad-spectrum antitumor effect by regulating key nodes such as the protein balance of the Bcl-2 family and caspase activation.^[18]^ This dual regulatory mechanism of the apoptotic pathway makes it have potential application value in overcoming the apoptosis resistance of tumor cells, especially for the types of malignant tumors that are prone to drug resistance in traditional treatments.

**Figure 13.**
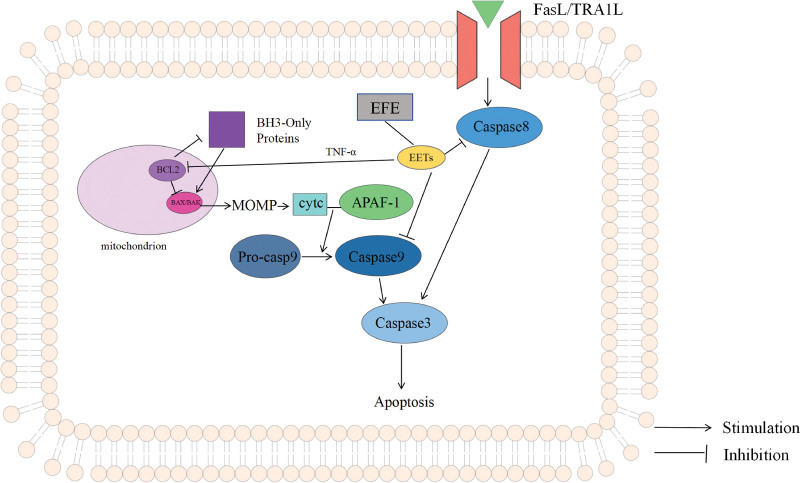
The mechanism of EFE through Bcl-2 in Caski cells. EFE = earthworm fibrinolytic enzyme.

In this study, EFE induced the decrease of bcl-2 expression in Caski cells, which may be related to the regulation of tumor necrosis factor (TNF) signaling pathway by affecting the metabolic pathway of arachidonic acid (AA).^[30]^ The AA metabolite epoxyeicosatrienoic acid (EETs) down-regulates the level of antiapoptotic protein Bcl-2 via TNF-α.^[31]^ In addition, the activity of caspase-8 and caspase-9 was significantly reduced, and the effect of antiapoptotic protein Bcl-2 was inhibited.^[32]^ The possible mechanism is shown in Figure 13.

Cyclin D1 protein is encoded by the proto-oncogene CCND1 as an important cell cycle driver, and its mutation, expansion and overexpression can change the cell cycle process and contribute to tumorigenesis, so it has a prognostic and predictive role in cancer.^[33]^ Cyclin D1 binds to and activates CDK4, a cyclin-dependent kinase specific to the G1 phase.^[34]^ The G1 cycle suppressor protein (Rb) is phosphorylated, and the phosphorylated Rb protein is dissociated from the E2F transcription factor to which it binds, which begins to transcribe the genes of the living cell cycle.^[35,36]^ This pushes the cell cycle from the G1 phase to the S phase, initiating mitosis. Moreover, NF-κB signaling plays a key role in tumor cell progression.^[37]^ Cyclin D1, c-myc, survivin and EMT-related proteins are the downstream intracellular signaling molecules of NF-κB signal transduction.^[38,39]^ Activated NF-κB translocates to the nucleus and regulates gene transcription to activate various downstream targets, such as activating the cell cycle regulator cyclin D1.^[40]^ Activated NF-κB directly binds to specific sequences in the cyclin D1 promoter, leading to upregulation of cyclin D1 expression and over-phosphorylation of pRb.^[41]^ This induces the cell cycle transition from G1 to S phase and increases cell proliferation.^[42,43]^

**Figure 14.**
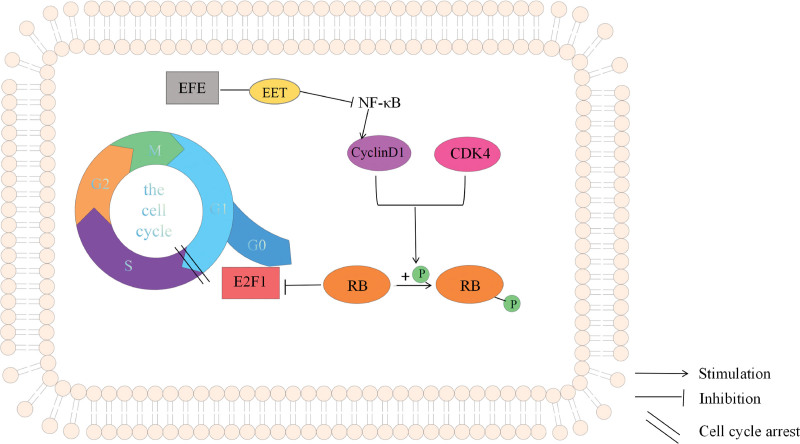
The mechanism of EFE through cyclin D1 in Caski cells. EFE = earthworm fibrinolytic enzyme.

In this study, EFE induced reduced cyclin D1 expression in Caski cells, which may be related to the regulation of NF-κB by affecting the metabolic pathway of AA.^[30]^ EET is an epoxy fatty acid derived from AA metabolism and has anti-inflammatory effects.^[44]^ EET down-regulates the expression of NF-κB target gene and then down-regulates the expression of Cyclin D1 protein.^[45]^ The possible mechanism is shown in Figure 14.

**Figure 15.**
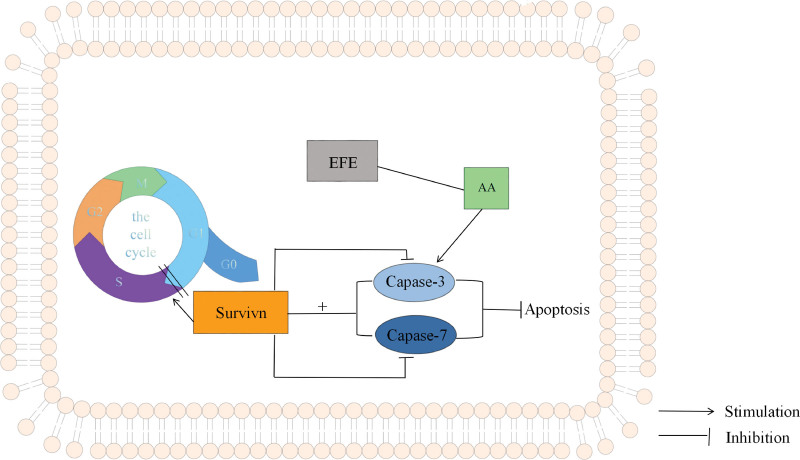
The mechanism of EFE through survivin in Caski cells. EFE = earthworm fibrinolytic enzyme.

Survivin is a potential tumor marker that plays a role in modulating tumor cell viability, anti-apoptosis, and amplifying tumor progression.^[46]^ Survivin is the smallest member of the apoptosis suppressor protein family, which has dual functions of inhibiting apoptosis and regulating cell division. The BIR domain of survivin contains amino acids that play an important role in apoptosis and specifically binds directly to the end-effect enzymes caspase-3 and caspase-7 downstream of the apoptosis signaling pathway, inhibit its activity and exert antiapoptotic effect.^[47]^ Survivin is one of the subunits of the chromosome passenger complex and is involved in mitotic microtubule polymerization, chromosome segregation, and cytoplasmic packing.^[48,49]^ In tumor cells, survivin overexpression inhibits G1 quiescence and promotes cell cycle transition to S phase, and survivin upregulation is detected in G2/M phase.^[50]^ Survivin protects the integrity of mitotic organelles by specifically binding to spindle tubules to prevent the spindle from being hydrolyzed, thus contributing to the continuous progression of mitosis and promoting cell proliferation. Overexpression of survivin is associated with hyperplasia and tumor and can be used as a biomarker for these diseases.^[51]^ The possible mechanism is shown in Figure 15.

In this study, EFE-induced reduction of survivin expression in Caski cells may be related to the regulation of caspase-3 by affecting the metabolic pathway of AA.^[30]^ AA is an essential fatty acid released by phospholipids in cell membranes. AA induces caspase-3 activation, thereby inhibiting survivin activity.^[52,53]^

EFE has achieved certain research results in breast cancer, colorectal cancer, and lung cancer. Therefore, combined with the latest experimental findings in cervical cancer, we systematically expounded the molecular mechanism of EFE’s multi-target antitumor and its clinical translational value.

In terms of the mechanism of action, EFE shows significant multi-pathway synergistic effects. Apoptosis induction is its most prominent antitumor mechanism: in breast cancer, it is mainly achieved by inhibiting the PI3K/AKT/mTOR signaling pathway^[54]^; in colorectal cancer, by activating caspase-3 and regulating the Wnt/β-catenin pathway^[55]^; in cervical cancer, it has been newly discovered that it metabolizes the TNF signaling axis through AA. On the one hand, it promotes the down-regulation of the expression of antiapoptotic protein bcl-2 by EETs. On the other hand, it activates caspase-3 through AA and thereby inhibits the expression of survivin.^[31,32]^ This multilevel apoptosis-inducing network enables EFE to have a good proapoptotic effect on different cancer types.

In terms of cell cycle regulation, EFE blocks the cell cycle through the ROS/JNK pathway in lung cancer,^[56]^ and in cervical cancer, it has been newly discovered that it effectively inhibits tumor cell proliferation by down-regulating the expression of cyclin D1, a key target gene of the NF-κB signaling pathway, through EETs.^[44,45]^ This cycle blocking effect forms a synergy with its proapoptotic effect and jointly inhibits tumor growth.

It is particularly notable that this study discovered for the first time in cervical cancer that EFE simultaneously affects 3 key pathological processes by regulating the AA metabolic network: regulating apoptosis through bcl-2/survivin, inhibiting proliferation through cyclin D1, and regulating the inflammatory microenvironment through NF-κB. This multi-target synergistic action pattern is highly consistent with the pathogenesis characteristics of HPV-related cervical cancer,^[57]^ because HPV carcinogenesis is precisely a multifactor process involving abnormal proliferation, apoptotic resistance, and chronic inflammation.

From the perspective of clinical transformation, the multi-target nature of EFE endows it with broad-spectrum antitumor potential, and its characteristic of functioning through metabolic regulation may reduce the risk of drug resistance. EFE has shown a synergistic effect with trastuzumab in breast cancer,^[58]^ and remains active against chemotherapy-resistant strains in colorectal cancer.^[59]^ These characteristics suggest that it may have significant value in the comprehensive treatment of cervical cancer. Especially for the problems currently faced in the treatment of cervical cancer, such as drug resistance to radiotherapy and chemotherapy and low response rate of immunotherapy, EFE may provide new solutions.

This study not only expands the understanding of the antitumor mechanism of EFE, but more importantly, provides a theoretical basis for the development of new cervical cancer treatment regimens based on metabolic regulation. In terms of mechanism research, it is necessary to deeply analyze the regulatory effect of EFE on HPV oncogenic proteins E6/E7, and comprehensively clarify the AA metabolic network it regulates by using multi-omics techniques. In terms of the optimization of treatment strategies, the synergistic effect of EFE and the existing standard treatments (cisplatin/radiotherapy/immunotherapy) should be systematically evaluated, and the combination medication regimens based on EFE should be developed to solve the problem of clinical drug resistance. In terms of formulation research and development, it is necessary to design sustained-release dosage forms suitable for local administration of the cervix (such as vaginal suppositories and gels) and develop nano-targeted delivery systems to increase the drug concentration at the tumor site. These studies will accelerate the transformation process of EFE from the laboratory to clinical application and provide new treatment options for patients with cervical cancer.

## 6. Conclusions

As a multitarget antitumor drug of natural origin, EFE has a unique metabolic regulation mechanism and good safety characteristics, which makes it have broad development prospects in the field of comprehensive tumor treatment. This study shows that different concentrations of EFE can inhibit the proliferation, migration of Caski cells, and promote apoptosis of cervical cancer cells, which laid a scientific basis for the treatment of clinical cervical cancer by EFE.

## Author contributions

**Conceptualization:** Kaize Yuan, Yiyao Shi, Ke Zhang.

**Data curation:** Kaize Yuan, Yiyao Shi.

**Formal analysis:** Kaize Yuan, Yiyao Shi, Ke Zhang.

**Funding acquisition:** Kaize Yuan, Siyang Chen, Yiyao Shi.

**Investigation:** Kaize Yuan, Siyang Chen, Miao Yu, Ke Zhang, Lihua Zhu.

**Methodology:** Kaize Yuan, Siyang Chen, Miao Yu, Lihua Zhu, Shuying Li.

**Project administration:** Kaize Yuan, Siyang Chen.

**Resources:** Kaize Yuan, Siyang Chen, Miao Yu, Ke Zhang, Lihua Zhu.

**Software:** Kaize Yuan, Yuehua Bai, Siyang Chen.

**Supervision:** Kaize Yuan, Yuehua Bai, Siyang Chen, Miao Yu, Ke Zhang, Shuying Li.

**Validation:** Kaize Yuan, Yuehua Bai, Yiyao Shi, Ke Zhang, Lihua Zhu, Shuying Li.

**Visualization:** Kaize Yuan, Yuehua Bai, Yiyao Shi, Miao Yu, Ke Zhang.

**Writing – original draft:** Kaize Yuan, Yuehua Bai, Yiyao Shi, Miao Yu.

**Writing – review & editing:** Kaize Yuan, Yiyao Shi.
